# Detection of autism spectrum disorder using graph representation learning algorithms and deep neural network, based on fMRI signals

**DOI:** 10.3389/fnsys.2022.904770

**Published:** 2023-02-02

**Authors:** Ali Yousefian, Farzaneh Shayegh, Zeinab Maleki

**Affiliations:** Department of Electrical and Computer Engineering, Isfahan University of Technology, Isfahan, Iran

**Keywords:** autism spectrum disorder, connectivity, graph representation learning methods, AWE, Graph2Img, deep neural network (DNN)

## Abstract

**Introduction:**

Can we apply graph representation learning algorithms to identify autism spectrum disorder (ASD) patients within a large brain imaging dataset? ASD is mainly identified by brain functional connectivity patterns. Attempts to unveil the common neural patterns emerged in ASD are the essence of ASD classification. We claim that graph representation learning methods can appropriately extract the connectivity patterns of the brain, in such a way that the method can be generalized to every recording condition, and phenotypical information of subjects. These methods can capture the whole structure of the brain, both local and global properties.

**Methods:**

The investigation is done for the worldwide brain imaging multi-site database known as ABIDE I and II (Autism Brain Imaging Data Exchange). Among different graph representation techniques, we used AWE, Node2vec, Struct2vec, multi node2vec, and Graph2Img. The best approach was Graph2Img, in which after extracting the feature vectors representative of the brain nodes, the PCA algorithm is applied to the matrix of feature vectors. The classifier adapted to the features embedded in graphs is an LeNet deep neural network.

**Results and discussion:**

Although we could not outperform the previous accuracy of 10-fold cross-validation in the identification of ASD versus control patients in this dataset, for leave-one-site-out cross-validation, we could obtain better results (our accuracy: 80%). The result is that graph embedding methods can prepare the connectivity matrix more suitable for applying to a deep network.

## 1. Introduction

Autism spectrum disorder (ASD) is a set of clinical presentations, emerging due to neurodevelopmental disorder. ASD symptoms are related to social communication, imagination, and behavior. Accurate and timely diagnoses of ASD significantly improve the quality of life of individuals with ASD (Elder et al., 2017). Yet, there is no clear etiology to diagnose ASD.

To date, ASD diagnosis is done based on the behavioral characteristics of children, observed by parents and teachers at home or school (Nickel and Huang-Storms, 2017; Almuqhim and Saeed, 2021). Since autism is related to abnormal development of the brain, assessing brain function (e.g., based on fMRI) is at the top of automatic diagnosis and classification research. Resting-state functional MRI has a suitable spatial resolution to show the interaction of brain regions during a special behavior. In other words, a region’s function is tightly dependent on its interactions. Various differential observations consider the properties of the brain network in healthy and ASD subjects. However, statistical analysis led the researchers to a disorder whose mechanisms vary among patients. In other words, there is no unique fact to announce it as a reliable biomarker of ASD (Frye et al., 2019).

By considering brain regions and their connections as a network, the detection of ASD alternatively could be a network classification task, in which machine learning techniques could help. To efficiently use information hidden in the resting-state fMRI, the connectivity measures obtained from resting-state fMRI are useful to understand the large-scale functional difference between healthy and abnormal brains.

After that, a suitable classifier should be used. A vast number of mental disorder diagnosis studies used traditional classifiers, such as support vector machine (SVM), LASSO, and Bayesian classifier. But deep learning methods showed a major preference in the case of connectivity matrix because it is a high-dimensional feature of brain activity. High-dimensional features increase the number of hyperparameters of a machine learning algorithm. In such a way, just deep neural networks can learn complex structures of high-dimensional data. From this point of view, applying fully connected deep neural networks and convolutional networks on fMRI volumes and raw connectome data appears to be successful (Heinsfeld et al., 2018; El Gazzar et al., 2019; Sherkatghanad et al., 2020).

In Heinsfeld et al. (2018), a deep learning algorithm using the full connectivity matrix is applied to classify ASD and controls using ABIDE data. They showed anterior–posterior underconnectivity in the autistic brain and surpassed the state-of-the-art classification of autism by achieving 70% accuracy. Similarly, a convolutional neural network (CNN) was used to effectively diagnose Alzheimer’s disease (AD) (Sarraf et al., 2016) and mild cognitive impairment (MCI) (Meszlényi et al., 2017). In another study, CNN was used to extract features from fMRI data, and SVM was used for classification (Nie et al., 2016). A deep autoencoder was used to classify the fMRI data of MCI (Suk et al., 2016). Furthermore, different hidden layers between the encoder and the decoder (Patel et al., 2016) were added to afford different tasks, like denoising (Heinsfeld et al., 2018), or generating sparse features (Guo et al., 2017). Other networks such as radial basis function network (RBFN) (Vigneshwaran et al., 2015), restricted Boltzmann machine (RBM) (Huang et al., 2016), and deep Boltzmann machine (DBM) can be used to extract features from fMRI data because they can combine the information of different voxels of the region of interest (Zafar et al., 2017). To take advantage of the topological information implied in the connectivity graph, a restricted path-based depth-first search (RP-DFS) algorithm was applied to some remarkable autistic functional connections (Huang et al., 2020). Finally, a three-layer deep belief network (DBN) model with the automatic hyperparameter-tuning technique was applied for classification. To date, this work achieved the most accurate ASD/healthy classification result for ABIDE dataset (76.4% accuracy).

However, to get more reliable results, dynamic and/or multimodal features were proposed. As an example, CNN with the wavelet-based spectrogram as input (instead of the static connectivity matrices), taking the dynamic of brain activities into account, reached a specific improvement in the classification accuracy (Al-Hiyali et al., 2021). However, just 144 subjects of the ABIDE database were used in their evaluation. Furthermore, a novel adversarial learning-based node–edge graph attention network (AL-NEGAT) is used to combine fMRI and structural MRI information (Chen et al., 2022) and obtained 74.7% accuracy. But this method could not reach a good result in leave-one-site-out validation (69.42%).

On the other hand, the benefit of DNN is mainly due to a large number of training examples (Kuang et al., 2014; Kim et al., 2016; Guo et al., 2017; Heinsfeld et al., 2018). Developing deep learning approaches to work with functional connectivity (FC) features using small or at best modest sample sizes of neurological data (di Martino et al., 2014; Kuang et al., 2014; Kim et al., 2016; Guo et al., 2017; Heinsfeld et al., 2018) is debatable from the reproducibility and generalizability point of view. One solution is the deep transfer learning neural network (DTL-NN) approach that could achieve improved performance in classification for neurological conditions (70.4% for ASD detection), especially where there are no large neuroimaging datasets available (Li et al., 2018). Other solutions are the Synthetic Minority Oversampling Technique (SMOTE) to perform data augmentation to generate artificial data and avoid overfitting (Eslami and Saeed, 2019) and sparse autoencoder (SAENet) that was used for classifying patients with ASD from typical control subjects using fMRI data (70.8% accuracy and 79.1% specificity) for the whole dataset as compared to other methods (Almuqhim and Saeed, 2021). Another approach is to develop a machine learning approach with a robust training methodology (Li et al., 2018). Machine learning algorithms able to extract replicable, and robust neural patterns from brain imaging data of patients with ASD, reach suitable classification results (Pereira et al., 2009).

Another solution in studies with limited sample sizes is the reduction of the size of features indicating useful connectivity properties by network analysis methods. The ease of representing brain connectivity information according to graph theory makes them very valuable tools in this area. Machine learning on graphs finds its importance here: finding a way to represent or encode graph structure is the subject of this task. Nowadays, in order to model information underlying the graph structure, there are new ways of representing and analyzing graphs, which afford the complexity of working with big graphs. Referring to these representation algorithms as embedding, applying these approaches to brain networks is named connectome embeddings (CEs). These embedding algorithms involve converting graphs into vectors. Network embedding techniques can be divided into three buckets: (1) based on engineered graph features, (2) obtained by training on graph data, and (3) obtained by a layer of a deep network. The main drawback of the former is that structural homologies or higher-order relations of the connectivity matrix could not be captured (Rosenthal et al., 2018). Furthermore, these features are not flexible; *i.e.*, they cannot adapt during the learning procedure. In summary, many of these local and global features cannot capture the topological shape of the graph, unless the morphology of the cortex would be considered (He et al., 2022).

In the second bucket, referred to as shallow embedding, network embedding vectors are learned by optimizing different types of objective functions defined as a mapping to reflect geometric information of graph data. This optimum embedded space is the final feature vector. These algorithms involve learning approaches that map nodes to an embedding space. Anonymous walk Embedding (AWE), Node2vec, Struct2vec, DeepWalk, multi-node2vec, and Graph2Img (Grover and Leskovec, 2016; Ribeiro et al., 2017) are some well-known algorithms of this bucket. These methods represent higher-order features of the connections of a graph, helpful to develop an input convenient for training a CNN. As an example, in the Graph2Img method, the embedded space of the brain network is transformed into an image. The advantage of this method is the capability of dimensionality reduction of this image by an algorithm like PCA and still has an image at hand (Meng and Xiang, 2018). Multi-node2vec was applied on fMRI scans over a group of 74 healthy individuals. Multi-node2vec identifies nodal characteristics that are closely associated with the functional organization of the brain (Wilson et al., 2018).

In the third bucket, referred to as deep embedding, CE and deep learning algorithms are combined to form a single deep network. This combinatory network can exploit the connectome topology. In this category, a Hypergraph U-Net (HUNet), Graph U-Net (GUNet) (Gao and Ji, 2019), and hypergraph neural network (HGNN) (Feng et al., 2019) are proposed in which low-dimensional embeddings of data samples are learned from the high-dimensional connectivity matrix. Indeed, these networks emerged as a subset of deep graph neural networks (GNNs) (Wang et al., 2016; Kipf and Welling, 2017; Gao and Ji, 2019) are able to model the deeply nonlinear relationship node connectomic features (Ktena et al., 2017; Banka and Rekik, 2019; Bessadok et al., 2019).

In summary, the accuracy of ASD classifiers using different algorithms ranges from 55 to 76.4% (Parisot et al., 2018; Xing et al., 2019; Huang et al., 2020; Kazeminejad and Sotero, 2020; Sharif and Khan, 2021; Chen et al., 2022). The main point is that the reported good performances in ASD classification of ABIDE dataset were about considering individual sites for most traditional and deep machine learning algorithms (Huang et al., 2020; Sherkatghanad et al., 2020). But our main concern is that after intermingling all the sites, or leave-one-site-out cross-validation algorithm, accuracy (the percent of correctly classified subjects), and the area under ROC is diminished. In other words, there is no algorithm appropriate for clinical usage. Thus, still, further experiments are required to be conducted with patients with different phenotypical information to ensure the clinical value of these methods (Li et al., 2018).

Our main goal in this paper is the demonstration of the role of the second bucket (CE method) in representing the structure with which brain regions are connected to each other and assessing its effect on ASD classification. In fact, we claim that representation-based features can solve the problem of high-dimensional input of the deep network. Based on the ABIDE I and ABIDE II public datasets, recorded at some different sites, we want to investigate whether CE can surpass previous research studies or not. Accordingly, by using CNN classifiers, we claim that there is great potential in combining graph representation methods, with deep learning techniques for fMRI-based classification, to increase the generalization of the algorithm from one site to others.

The structure of the paper is as follows: after describing the network embedding techniques in Section “2. Materials and methods,” suitable embedding-based features are illustrated. In Section “3. Implementation and results,” the classification technique using the deep network is declared. Afterward, ABIDE database, its preprocessing methods, and the embedded features extracted from them are introduced. These features are applied to deep network to detect ASD subjects. Some evaluation measures like the F-score and the accuracy of this classifier are reported in the Results section and are compared to other literature working on ABIDE dataset.

## 2. Materials and methods

### 2.1. Network embedding methods

The concept of network embedding can be described as follows: suppose there is a graph *G* = (*V*,*E*,*A*) with *V* as the node set, *E* as the undirected and weighted edge set, and *A* as the adjacency matrix. We are going to find the optimum function *z* = *f*(*v*) ∈ *R^d^* that maps each node or subgraph to a d-dimensional vector disclosing the structure of the graph. These vectors should be representative of the graph and can be used as the feature vectors uncovering the similarities of the graph for machine learning algorithms. At this level, each node corresponds to a d-dimensional embedded vector involving its connections with all other nodes (Hamilton William et al., 2017).

Indeed, these low-dimensional embedded vectors can summarize either position of nodes in the graph, or the structure of their neighborhood, and user-specified graph statistics (Hamilton William et al., 2017). Most shallow embedding mapping techniques are done based on a lookup table, just like what occurred in classic matrix factorization for dimensionality reduction (Hamilton William et al., 2017). For another part of shallow embedding techniques, learning the embedded vector for each node is the process of training an encoder–decoder system, defined as an optimization method. The decoder maps the similarity of two nodes into a real-valued similarity measure. Different techniques able to afford this job (like DeepWalk, Node2vec, AWE, TSNE, GraRep, and others) are based on a stream of randomly generated walks. The resultant vectors can describe the similarities and subgraph membership with relatively few dimensions. These learned embedded vectors can be used as features of the graph.

The core of this relevant optimization problem is to find a mapping such that nearby nodes in short random walks have similar embedding vectors. The detail of random walk, DeepWalk, and Node2vec embedding methods is explained in Supplementary Appendix A.

#### 2.1.1. Struc2vec

Node2vec and DeepWalk approaches lead to a unique embedding vector for every individual node but have some drawbacks, including working as a lookup table, its computational cost, failure to leverage attribute information of nodes involving node’s position and role, weakness in predicting information of unseen nodes. To alleviate the abovementioned drawbacks, two alternatives have arisen: (1) some embedding approaches that enable capturing the structural roles of nodes have been proposed (Ribeiro et al., 2017; Donnat et al., 2018), and (2) network embedding in a feature-based manner has been proposed.

As an example of the first alternative, Ribeiro’s technique referred to as Struc2Vec generates some new graphs *G*_0_, … , *G*_*K*_, each to capture one kind of *k*-hop neighborhood structural similarity, from the original graph *G*. The algorithm is as follows (Ribeiro et al., 2017):

1. For each node *v_i_*, order the sequence of degrees of nodes exactly with the distance of *k*-hops from it: *R*_*k*_(*v*_*i*_).
2. Start from a weighted graph *G_0_* whose edges have zero weights *w*_0_(*v*_*i*_,*v*_*j*_)=0.
3. Build a sequence of weighted graphs whose edges vary adaptively by the equation:

$$w_{k}\;\left(v_{i},v_{j}\right)=w_{k-1}\;\left(v_{i},v_{j}\right)+d\;\left(R_{k}\;\left(v_{i}\right),R_{k}\;\left(v_{j}\right)\right)$$

where *d* (*R*_*k*_ (*v*_*i*_) , *R*_*k*_ (*v*_*j*_)) is the distance between sequence *R*_*k*_ (*v*_*i*_) and *R*_*k*_ (*v*_*j*_) and could be defined with different measures.
4. Run random walk on new graphs *G*_0_,…,*G*_*K*_ to implement Node2vec on them, and learn latent from them, using an algorithm like SkipGram.

In the second alternative, namely, feature-based methods, two algorithms referred to as Graph2Img and AWE are considered.

#### 2.1.2. Graph2Img

The Graph2Img algorithm, at first, transfers the original network into feature vectors and then uses clustering methods to group nodes. In other words, after embedding the graph nodes into a *d*-dimensional space, representations of nodes are gathered in a matrix of dimension *N*×*d*, where *N* = |*V*|, *i.e.*, the number of nodes in the graph. Next, we can decide whether all features are important or not and determine their priority. In fact, the principal component analysis (PCA) method is used to reduce the*d*-dimension vector to *dPCA*-dimension. Then, we can use just the most important dimension, the second important, third, and fourth dimensions. Taking into account just four first components of LPCA, two matrices *M*_*12*_ and *M*_*34*_ are constructed (see Supplementary Appendix C), which seems to be enough to analyze the brain network.

**FIGURE 1:**
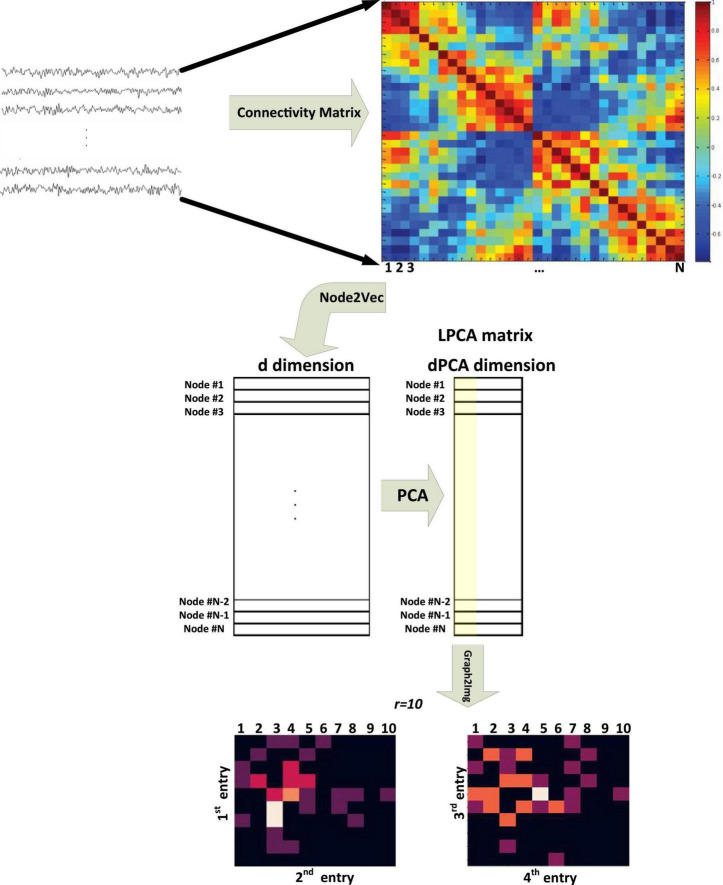
Block diagram of the Graph2Img feature-based embedding algorithm (Meng and Xiang, 2018).

As shown in Figure 1 (Meng and Xiang, 2018), these two matrices, behaving like images, can be applied as different channels of DCNN. The algorithm pseudo-code is shown in Supplementary Algorithm A-3 (Meng and Xiang, 2018).

#### 2.1.3. Anonymous walk embedding (AWE)

As another feature-based network embedding method, the Anonymous walk Embedding (AWE) algorithm used distribution of anonymous walks. Anonymous walks are the set of walks starting from an initial node *u*, by length *l* passing from random nodes, and termination at node *v*. There are a set of η such random walks $A_{l}^{u}=\left(a_{1}^{u},a_{2}^{u},\ldots ,a_{\eta }^{u}\right)$. Thus, the number of all possible random walks with length *l* exponentially grows with *l*. These anonymous walks capture structural information of nodes because labels of the nodes constituent of a random walk are omitted for them. In fact, corresponded to the random walk: *w* = (*v*_1_,*v*_2_,…,*v*_*k*_), we can define an anonymous walk involving a sequence of integers *a* = (*f*(*v*_1_),*f*(*v*_2_),…,*f*(*v*_*k*_)) where *f*(*v*) is the minimum place of *v* in the *w* random walk (Ivanov and Burnaev, 2018). However, due to the huge number of anonymous walks of a large graph, an efficient sampling approach is required to approximate this distribution (Ivanov and Burnaev, 2018). Defining the objective function of similar nodes on local neighborhoods of anonymous walks, improve the structural consideration of the embedding method.

These four embedding algorithms, Node2Vec, DeepWalk, AWE, and Graph2Img, extract the feature vectors of each node, describing the characteristics and structure of the graph. Thus, the next step of our research is the classification of these feature vectors obtained for healthy and ASD subjects.

### 2.2. Classification

Graph classification is a task to predict whether a whole graph belongs to any class of *C* predefined classes. In other words, the task is to train a classifier based on *N* graphs {*G*_*i*_},*i* = 1:*N* and their corresponding labels {*L*_*i*_},*i* = 1:*N*, able to classify every new graph *G*→*L*. Graph classification problem can be done using two typical approaches: (1) classification using extended CNNs to be appropriate for the raw graphs (Niepert et al., 2016) and (2) graph kernel methods (Shervashidze et al., 2011), in which graph embeddings *f*(*G*_1_) are used in conjunction with kernel methods (*K*(*f*(*G*_1_),*f*(*G*_2_))) to perform classification of new graphs, where *K*:(*x*,*y*)→*R^n^* is a kernel function, quantifying the distance of graphs.

As mentioned earlier, the aim of this paper is a kernelized classification of healthy and autistic patients based on functional connectivity matrices. The features extracted from these matrices (*f*(*G*_1_)) are the embedded vectors obtained by using Node2vec, Struct2vec, AWE, and Graph2Img algorithms. To do the classification job, we used the DNN classifier. The reason underlying this selection is the size of the resultant feature vectors, whose classification requires many parameters to be trained. As well, to validate the performance of our classification task, cross-validation is applied.

Three types of deep networks have been considered in this study: LeNet, ResNet, and VGG16. However, finally, we have used LeNet, because of its best performance for our problem. Thus, we described it here.

#### 2.2.1. LeNet

*LeNet*, one of the first published CNNs in computer vision tasks, was introduced by (and named for) Yann LeCun. In LeCun et al. (1998) published the first study in which he could train CNNs *via* backpropagation. Then, this network was applied in AT&T Bell Labs, for the purpose of recognizing handwritten digits in images (LeCun et al., 1998). LeNet achieved outstanding results comparable with that of support vector machines and thus became a dominant approach in supervised learning.

**FIGURE 2:**
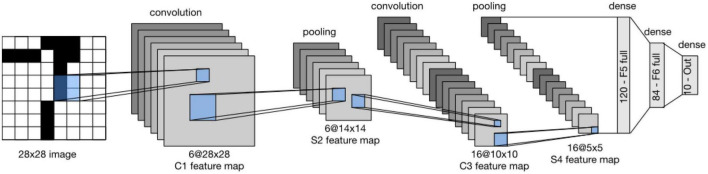
Data flow in LeNet. The input is an image, and the output is a probability over different possible outcomes (Loey et al., 2016).

LeNet (LeNet-5) consists of two parts (Wang and Gong, 2019): *(i)* a convolutional encoder and *(ii)* a dense block. The former consists of two convolutional blocks, and the latter consists of three fully connected layers. The architecture is summarized in Figure 2.

Each convolutional block includes a convolutional layer, a sigmoid activation function, and a subsequent average pooling operation. In 1990, ReLUs and max pooling were discovered to have suitable performance. However, in LeNet, each convolutional layer maps any 5 × 5 part of the input to a scalar using a kernel and a sigmoid activation function. There are 6 convolutional layers, in such a way that the result is a 6@28*28 tensor. In fact, by these convolutional layers, spatial features of input are mapped to a number of two-dimensional feature maps, namely, channels. Then, a pooling layer samples the channels by a factor of 2 and leads to a 6@14*14 array. Then, there is another convolutional layer. Again, this is a convolutional layer with a 5*5-dimensional filter. The first convolutional layer had 6 output channels, while the second layer has 16 outputs of size 10*10. The output of the convolutional block must be flattened before being passed to the dense block. This output is a 16@5*5 vector, created by a pooling layer.

LeNet’s dense block has three fully connected layers, with 120, 84, and 10 outputs, respectively. Because we are still performing classification, the 10-dimensional output layer corresponds to the number of possible output classes. Implementing LeNet models with modern deep learning frameworks is remarkably simple.

### 2.3. ABIDE dataset

The rs-fMRI data of ASD and healthy subjects are downloaded from a large multisite data repository Autism Brain Imaging Data Exchange (ABIDE)^1^. The Autism Brain Imaging Data Exchange I (ABIDE I) is a multisite platform gathered from 17 international laboratories, which shared some collected resting-state functional magnetic resonance imaging (rs-fMRI), anatomical and phenotypic datasets. This dataset includes 1112 patients, from 539 individuals with ASD and 573 from typical controls (age 7–64 years, median 14.7 years across groups). Till now, these data are used in many research studies. The publications have shown its utility for capturing the whole brain and regional properties of the brain connectome in ASD. All data have been anonymized.

Accordingly, ABIDE II was established to further promote discovery science on the brain connectome in ASD. To date, ABIDE II involves 19 sites, overall donating 1114 datasets from 521 individuals with ASD and 593 controls (age range: 5–64 years). All datasets are anonymous, with no protected health information included.

There is no ASD/healthy label for some individuals present in ABIDE database. After removing these cases, 871 individuals of ABIDE I and 910 individuals of ABIDE II would be remaining, for investigation in this study (Yang et al., 2019).

## 3. Implementation and results

**FIGURE 3:**
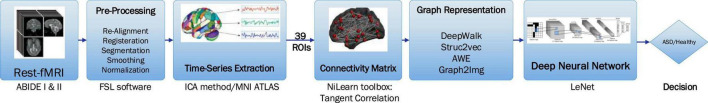
The steps of the proposed method, including the preprocessing, the 39 ROIs, the connectivity matrix, graph representation methods, and the deep learning classification.

The proposed method includes preprocessing, extracting the connectivity matrix, graph representation methods, and the deep learning classification. These steps are schematically shown in Figure 3.

### 3.1. Preprocessing and connectivity matrix

The rs-fMRI data are slice time corrected, motion corrected, registered, and normalized, using FSL software. The steps of preprocessing done for ABIDE I and ABIDE II databases are as follows: (1) AC-PC realignment, (2) gray matter and white matter tissue segmentation, (3) nonlinear registration to MNI152 space, (4) normalization, (5) resampling, (6) modulation, and (7) smoothing with FWMH = 4 mm. For the task of brain parcellation, the ICA method is used (de Martino et al., 2007; Tohka et al., 2008; Smith et al., 2009; Joel et al., 2011). In other words, instead of obtaining the average of the time series (BOLD signal) of some predefined regions, spatial maps output from ICA with the specific functional and anatomical interpretation (the locations of brain tissue acting synchronously and with the same activity pattern) is taken into account. ICA is a data-driven model, which uses no *a priori* information about the brain and has been a popular approach in the analysis of fMRI data (Salimi-Khorshidi et al., 2014). In this study, ICA decomposed the whole BOLD fMRI data into 39 regions according to MNI ATLAS.

Afterward, the BOLD signal of these 39 ROIs is considered to compute their connectivity measures, by statistical measures such as Pearson correlation, partial correlation (Saad et al., 2009), and tangent correlation (Dadi et al., 2019). The size of the connectivity matrix is 39*39, according to the number of ROIs. The Pearson correlation coefficient ranges from 1 to −1, where 1 indicates that two ROIs are highly correlated, and −1 indicates that two ROIs are anticorrelated. This step is done using the Nilearn toolbox developed by MIT University, as well as the BrainIAK toolbox (Kumar et al., 2020). Nilearn is a python toolbox for statistical learning on neuroimaging data. In this study, the connectivity matrix is obtained *via* tangent correlation (Pedregosa et al., 2011). See Supplementary Appendix A for more details. This method is less frequently used but has solid mathematical foundations, and a variety of groups have reported good decoding performances with this framework. Connectivity matrices built with tangent space parametrization give an improvement compared to full or partial correlations.

### 3.2. Classifying the graph embedding vectors

**FIGURE 4:**
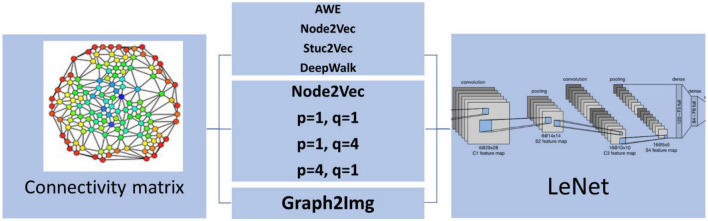
Three scenarios of applying embedding vectors to detect ASD *via* LeNet.

According to the abovementioned embedding features, we used three scenarios to check whether ASD detection can be improved by graph embedding algorithms or not. In the first scenario, features are embedded vectors of the connectivity matrix using each of the Node2Vec, Struc2Vec, and AWE methods. Accordingly, a deep network with one channel input is used in this scenario. This channel input is an *d*×*N* matrix including the *d*-dimensional embedded vectors of all *N* = 39 nodes. The embedded vectors obtained by these methods have a dimension of *d* = 25, 64, and 128, respectively. But, in the second scenario, to take different properties of the Node2vec algorithm, with different *p* and *q* values (*p* = 1,*q* = 1, *p* = 1,*q* = 4, and *p* = 4,*q* = 1), into account, a three-channel deep network is applied. In the third scenario, after applying PCA on the result of the Node2Vec algorithm, two matrices of the Graph2Img algorithm are considered as input of a two-channel CNN. These three scenarios are schematically shown in Figure 4.

Indeed, at first, we tried to do the classification job through traditional kernel-based classifiers, like support vector machine (SVM), but satisfactory results could not be obtained. The classifier could not show an accuracy better than chance. The advantage of CNN is that it is composed of an automatic feature extractor that again extracts features from the embedded vectors and, thus, is a trainable classifier.

In all three scenarios, we customize the LeNet structure for our problem: In the first scenario, there is one channel in the input layer, and the size of the embedded vector in each of the Node2vec, Struct2vec, and the AWE method determines the dimension of the input. These sizes are, respectively, equal to 25, 64, and 128.

Thus, in the first scenario, the input layer of LeNet is a 39*d,*d* = 25,64,128 image. In the second scenario, the network has three channels. Each channel of the deep network consists of 39*d neurons. In the third scenario, there are two channels, each consisting of 10*10 neurons (*r* = 10). Finally, in all three scenarios, there are two output neurons indicating a healthy and autism brain.

The default LeNet network was modified according to the abovementioned dimensions of input/output. Furthermore, a dropout layer is employed for regularization at every hidden layer [33] with 0.8 keeping regularity. Another difference is the activation functions we used in LeNet are ReLU functions, except for the ultimate layer, which uses a softmax function in such a way that a probability distribution over classes would be obtained. For the convolution pooling block, we employ 64 filters at the first level, and as the signal is halved through the (2,2) max pooling layer, the number of filters in the subsequent convolutional layer is increased to 96 to compensate for the loss in resolution (Tixier et al., 2019). The number of trainable weights in this deep neural network doubles or triples in the third and second scenarios.

The illustrated networks are used as the healthy/ASD classifier. Classification results would be reported in Supplementary Appendix to compare them with previous research in which a deep network is used to classify the raw connectivity matrices.

### 3.3. Evaluation

To check the performance of our proposed ASD classifier working based on graph embedding techniques and deep machine learning methods, two kinds of cross-validation techniques are used. Indeed, these two techniques depend on how we choose training and test datasets. According to the properties of ABIDE database that consists of different sites, we can do three different partitioning jobs: (1) dividing data of each site into *N* folds, and reporting accuracy of classification in individual sites, (2) leave-one-site-out validation (distinctly for ABIDE I and II), and (3) dividing all data of ABIDE I and II into *N* folds to report typical *N*-fold cross-validation. In all three approaches, the classification performance is assessed by accuracy, F-score, recall, and precision. To report the accuracy of all data, statistically more reliable, the second approach, i.e., leave-one-site-out validation, is the most appropriate one. However, in this paper, the validation types (2) and (3) are considered in the report of the results.

Considering ASD detection as the goal of the classifier, true positive (TP) is defined as the percent of ASD subjects correctly classified as ASD. As well, the percent of ASD subjects classified as healthy is referred to as false negative (FN). Similarly, false positive (FP) is the percentage of healthy subjects decided to be ASD. Accordingly, the F-score measure is defined as follows:


$$F-s\,c\,o\,r\,e=\frac{T\,P}{T\,P+\frac{1}{2}\,\left(F\,P+F\,N\right)}$$

It is important for the classifier to detect all ASD subjects, so $\frac{T\,P}{\#\,A\,S\,D\,s\,u\,b\,j\,e\,c\,t\,s}$ is referred to as recall. Also, it is expected for a classifier to have trusted positive detection, or in other words to be precise. Thus, precision is defined as $\frac{T\,P}{T\,P+F\,P}$. Because the size of subjects of two classes is not necessarily balanced, precision is a better measure of performance. Accordingly, another definition of F-score is based on recall and precision:


$$F-s\,c\,o\,r\,e=\frac{2\times r\,e\,c\,a\,l\,l\times p\,r\,e\,c\,i\,s\,i\,o\,n}{r\,e\,c\,a\,l\,l+p\,r\,e\,c\,i\,s\,i\,o\,n}$$

At last, to check how many subjects are correctly labeled, accuracy is a well-known measure.


$$A\,c\,c\,u\,r\,a\,c\,y=\frac{T\,P+T\,N}{a\,l\,l\,s\,u\,b\,j\,e\,c\,t\,s}$$

On the other hand, the time cost of training the classifier is another measure of the method under evaluation.

## 4. Results

Results of three scenarios for ABIDE I and ABIDE II database are presented in Table 1, using the LeNet classifier. In the results of Table 1, validation of type 3 is considered: all subjects of each database are taken into account, and then 5-fold and 10-fold cross-validations are applied. The average accuracy of these folds is reported for each scenario. Scenario 2 achieved the best performance in which a mean classification accuracy of 64% (recall 0.77%, precision 0.73%) and 66% (recall 80%, precision 80%) is obtained for ABIDE I and ABIDE II, respectively (in 10-fold cross-validation). The range of accuracy values was between 52 and 69% in individual folds. Based on the literature, this is not better than Heinsfeld et al. (2018), Huang et al. (2020), and Sherkatghanad et al. (2020) in which 70.22, 70, and 76.4% accuracies are reported.

**TABLE 1:** The 5- and 10-fold cross-validation results using different embedding methods and CNN classifier (LeNet).

	Method	ABIDE II (5-fold)	ABIDE I (5-fold)	ABIDE II (10-fold)	ABIDE I (10-fold)
Scenario 1	Struct2vec	54%	56%	56%	58%
Scenario 1	DeepWalk	55%	55%	56%	59%
Scenario 1	Node2vec *p* = 1,*q* = 4	59%	57%	62%	62%
Scenario 1	AWE	56%	58%	63%	65%
Scenario 2	Node2vec	63%	64%	66%	64%
Scenario 3	Graph2Img	59%	61%	66%	64%

The results of Table 1 show that the type of embedded features is effective in classification. But, as mentioned before, not given here, the results of SVM using embedded features are not better than those of Sherkatghanad et al. (2020), in which raw connectivity matrix has been used in classification *via* SVM. In other words, it seems that it is the art of deep network classifier in reaching (if any) good separation between ASD and healthy subjects, not the embedding features. So, the question is whether the feature embedding method was effective in ASD/healthy discrimination or not.

To answer this question, the results of the leave-one-site-out cross-validation are reported in Tables 2–5, respectively, for scenario 1 using AWE, scenario 2 using Node2vec, and scenario 3 using Graph2Img. In this validation type, just AWE of scenario 1 is applied, due to its better performance in the k-fold cross-validation procedure, against other embedding techniques. For each site, the LeNet CNN classifier is trained by data of other sites in each database and has been tested on data of that site. Results of the ABIDE I and ABIDE II are distinctly presented. The number of subjects at each site, number of ASD subjects, accuracy, and F-score of the proposed techniques, as well as those of Heinsfeld et al. (2018), Huang et al. (2020), and Sherkatghanad et al. (2020) (just for ABIDE I), are reported in the tables.

**TABLE 2:** Leave-site-out cross-validation results using scenario 1 just by AWE and CNN classifier.

	Sites	# Subjects	# ASD subjects	Accuracy	F-score	Accuracy (Heinsfeld et al., 2018)	F-score (Heinsfeld et al., 2018)	Accuracy (Sherkatghanad et al., 2020)	F-score (Sherkatghanad et al., 2020)	Accuracy (Huang et al., 2020)
ABIDE I	UCLA-2	21	11	0.90	0.86	0.66	0.65	0.61	0.62	0.78
	TRINITY	49	25	0.89	0.88	0.65	0.64	0.61	0.62	0.76
	UM-2	35	13	0.79	0.73	0.64	0.66	0.66	0.56	0.77
	KKI	33	12	0.92	0.91	0.67	0.66	0.72	0.69	0.79
	YALE	41	22	0.71	0.61	0.64	0.63	0.69	0.65	0.81
	PITT	50	24	0.76	0.73	0.66	0.65	0.69	0.73	0.78
	OLIN	28	14	0.77	0.73	0.64	0.63	0.58	0.56	0.76
	LEUVEN-2	28	12	0.79	0.76	0.65	0.64	0.65	0.73	0.81
	STANFORD	25	12	0.69	0.62	0.66	0.65	0.48	0.09	0.79
	NYU	172	74	0.76	0.73	0.66	0.65	0.65	0.73	0.74
	UM-1	86	14	0.71	0.67	0.64	0.63	0.66	0.56	0.77
	UCLA-1	64	37	0.74	0.72	0.66	0.65	0.69	0.65	0.78
	OHSU	25	12	0.82	0.77	0.64	0.64	0.57	0.56	0.73
	MAX-MUN	46	19	0.79	0.78	0.68	0.67	0.46	0.48	0.67
	LEUVEN-1	28	14	0.65	0.61	0.65	0.64	0.65	0.71	0.81
	USM	67	43	0.71	0.68	0.64	0.63	0.77	0.69	0.85
	SBL	26	12	0.72	0.67	0.66	0.65	0.56	0.62	0.66
	SDSU	36	14	0.86	0.81	0.63	0.63	0.75	0.80	0.80
	Mean			0.77	0.73	0.65	0.64	0.63	0.61	0.78
ABIDE II	BNI	56	29	0.79	0.77					
	EMC	54	25	0.83	0.82					
	ETH	37	13	0.82	0.65					
	GU	106	51	0.72	0.71					
	IP	54	21	0.81	0.80					
	IU	40	20	0.91	0.88					
	KKI	211	56	0.85	0.84					
	NYU	77	48	0.65	0.61					
	OHSU	93	37	0.72	0.69					
	ONRC	59	24	0.79	0.79					
	SDSU	58	33	0.79	0.73					
	SU	37	21	0.78	0.75					
	TCD	42	21	0.71	0.69					
	UCD	31	18	0.74	0.68					
	UCLA	32	16	0.85	0.82					
	USM	33	17	0.82	0.79					
	Mean			0.78	0.75					

**TABLE 3:** Leave-site-out cross-validation results using scenario 2 (Node2vec) and CNN classifier.

	Sites	# Subjects	# ASD subjects	Accuracy	F-score	Accuracy (Heinsfeld et al., 2018)	F-score (Heinsfeld et al., 2018)	Accuracy (Sherkatghanad et al., 2020)	F-score (Sherkatghanad et al., 2020)	Accuracy (Huang et al., 2020)
ABIDE I	UCLA-2	21	11	0.76	0.68	0.66	0.65	0.61	0.62	0.78
	TRINITY	44	25	0.72	0.64	0.65	0.64	0.61	0.62	0.76
	UM-2	34	13	0.71	0.63	0.64	0.66	0.66	0.56	0.77
	KKI	33	12	0.66	0.64	0.67	0.66	0.72	0.69	0.79
	YALE	41	22	0.81	0.76	0.64	0.63	0.69	0.65	0.81
	PITT	50	24	0.76	0.74	0.66	0.65	0.69	0.73	0.78
	OLIN	28	14	0.87	0.83	0.64	0.63	0.58	0.56	0.76
	LEUVEN-2	28	12	0.78	0.70	0.65	0.64	0.65	0.73	0.81
	STANFORD	25	12	0.82	0.82	0.66	0.65	0.48	0.09	0.79
	NYU	172	74	0.63	0.58	0.66	0.65	0.65	0.73	0.74
	UM-1	86	14	0.67	0.63	0.64	0.63	0.66	0.56	0.77
	UCLA-1	64	37	0.65	0.54	0.66	0.65	0.69	0.65	0.78
	OHSU	25	12	0.77	0.72	0.64	0.64	0.57	0.56	0.73
	MAX-MUN	46	19	0.60	0.53	0.68	0.67	0.46	0.48	0.67
	LEUVEN-1	28	14	0.85	0.82	0.65	0.64	0.65	0.71	0.81
	USM	67	43	0.76	0.69	0.64	0.63	0.77	0.69	0.85
	SBL	26	12	0.76	0.70	0.66	0.65	0.56	0.62	0.66
	SDSU	27	14	0.73	0.65	0.63	0.63	0.75	0.80	0.80
	Mean			0.73	0.68	0.65	0.64	0.63	0.61	0.78
ABIDE II	BNI	56	29	0.66	0.60					
	EMC	54	25	0.80	0.77					
	ETH	34	13	0.74	0.65					
	GU	106	51	0.64	0.59					
	IP	54	21	0.74	0.69					
	IU	34	20	0.75	0.67					
	KKI	34	56	0.79	0.70					
	NYU	77	48	0.68	0.61					
	OHSU	93	37	0.63	0.56					
	ONRC	49	24	0.80	0.75					
	SDSU	58	33	0.67	0.58					
	SU	54	21	0.70	0.64					
	TCD	39	21	0.76	0.74					
	UCD	31	18	0.77	0.72					
	UCLA	32	16	0.80	0.76					
	USM	33	17	0.70	0.64					
	Mean			0.72	0.66					

**TABLE 4:** Leave-site-out cross-validation results using scenario 3 (Graph2Img) and CNN classifier.

	Sites	# Subjects	# ASD subjects	Accuracy	F-score	Accuracy (Heinsfeld et al., 2018)	F-score (Heinsfeld et al., 2018)	Accuracy (Sherkatghanad et al., 2020)	F-score (Sherkatghanad et al., 2020)	Accuracy (Huang et al., 2020)
ABIDE I	UCLA-2	21	11	0.86	0.82	0.66	0.65	0.61	0.62	0.78
	TRINITY	44	25	0.79	0.75	0.65	0.64	0.61	0.62	0.76
	UM-2	34	13	0.78	0.72	0.64	0.66	0.66	0.56	0.77
	KKI	33	12	0.85	0.81	0.67	0.66	0.72	0.69	0.79
	YALE	41	22	0.82	0.81	0.64	0.63	0.69	0.65	0.81
	PITT	50	24	0.76	0.72	0.66	0.65	0.69	0.73	0.78
	OLIN	28	14	0.85	0.81	0.64	0.63	0.58	0.56	0.76
	LEUVEN-2	28	12	0.78	0.72	0.65	0.64	0.65	0.73	0.81
	STANFORD	25	12	0.94	0.92	0.66	0.65	0.48	0.09	0.79
	NYU	172	74	0.61	0.58	0.66	0.65	0.65	0.73	0.74
	UM-1	86	14	0.76	0.71	0.64	0.63	0.66	0.56	0.77
	UCLA-1	64	37	0.76	0.72	0.66	0.65	0.69	0.65	0.78
	OHSU	25	12	0.92	0.90	0.64	0.64	0.57	0.56	0.73
	MAX-MUN	46	19	0.77	0.71	0.68	0.67	0.46	0.48	0.67
	LEUVEN-1	28	14	0.82	0.78	0.65	0.64	0.65	0.71	0.81
	USM	67	43	0.78	0.73	0.64	0.63	0.77	0.69	0.85
	SBL	26	12	0.76	0.71	0.66	0.65	0.56	0.62	0.66
	SDSU	27	14	0.86	0.76	0.63	0.63	0.75	0.80	0.80
	Mean			0.80	0.76	0.65	0.64	0.63	0.61	0.78
EMCABIDE II	BNI	56	29	0.66	0.60					
	EMC	54	25	0.80	0.77					
	ETH	34	13	0.74	0.65					
	GU	106	51	0.64	0.59					
	IP	54	21	0.74	0.69					
	IU	34	20	0.75	0.67					
	KKI	34	56	0.79	0.70					
	NYU	77	48	0.66	0.61					
	OHSU	93	37	0.63	0.56					
	ONRC	49	24	0.80	0.75					
	SDSU	58	33	0.67	0.58					
	SU	54	21	0.70	0.64					
	TCD	39	21	0.76	0.74					
	UCD	31	18	0.77	0.72					
	UCLA	32	16	0.80	0.76					
	USM	33	17	0.70	0.64					
	Mean			0.72	0.66					

**TABLE 5:** Summary of best performance values and computational time for ABIDE I, in comparison to literature.

	Mean accuracy (10-fold cross-validation)	Mean accuracy (leave-one-site-out)	Computation time
Heinsfeld et al. (2018)	70%	65%	Over 32 h
Sherkatghanad et al. (2020)	70.22%	63%	12 h 30 min
Huang et al. (2020)	76.4%	78.2%	96 s
Chen et al. (2022)	74.7%	69.42%	−
Proposed method	66%	80%	2 h 47 min 20 s

**FIGURE 5:**
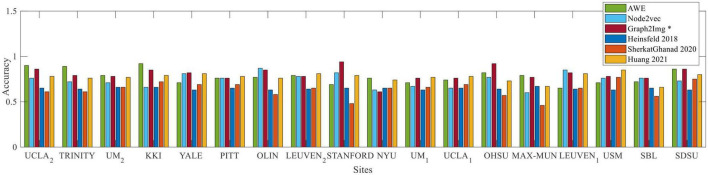
Box plot of leave-out-site accuracy to compare different embedding scenarios and Heinsfeld et al. (2018); Huang et al. (2020); Sherkatghanad et al. (2020) *vs*. sites, for ABIDE I dataset.

As shown in Figure 5, compared to Heinsfeld et al. (2018), Huang et al. (2020), and Sherkatghanad et al. (2020), which achieved the best results in the literature so far, the results depict that the Graph2Img-based CNN can outperform the other supervised methods. From this point of view, these results are in favor of embedding features, not just the deep network.

Therefore, some points worth considering in the results of these two validation methods:

1. Embedding features could not improve the results of the k-fold cross-validation but is able to improve the results of leave-one-site-out one.
2. The accuracy of CNN in classifying ASD subjects of each site is different when using graph embedding methods. On average, all embedding scenarios could improve the results, in comparison to using the raw connectivity matrices, in the leave-site-out validation manner (Heinsfeld et al., 2018; Sherkatghanad et al., 2020). The best embedding technique seems to be Graph2Img that increases the 65% (Heinsfeld et al., 2018) and 63% (Sherkatghanad et al., 2020) results to 80%. In our studies, the belief network of Huang et al. (2020) with 78.2% mean accuracy is the main rival of Graph2Img from the leave-one-site-out validation point of view that it also works based on embedding features, as well as a graph-based feature selection method.
3. Each graph embedding scenario has significantly improved the results of some sites, but not all of the sites.
• The AWE technique is not successful in the fMRI data of the University of Utah School of Medicine (USM), for which Huang et al. (2020) and Sherkatghanad et al. (2020) act well. For YALE University (YALE), the University of Leuven (LEUVEN), Stanford University (STANFORD), the University of Michigan (UM-1), and the University of California, Los Angeles (UCLA-1), Huang et al. (2020) reached better results than AWE.
• As well, Heinsfeld et al. (2018) and/or Sherkatghanad et al. (2020) outperform the embedding scenario 2 (three-channel node2vec with three values of *p* and *q*) in Kennedy Krieger Institute, Baltimore (KKI) data, New York University Langone Medical Center (NYU), Ludwig Maximilian University Munich (MAX-MUN), USM, and San Diego State University (SDSU) data. Almost at all sites of ABIDE I, scenario 2 reached less accuracy, in comparison to Huang et al. (2020), except Olin, Institute of Living, Hartford Hospital (OLIN), STANFORD, Oregon Health and Science University (OHSU), LEUVEN, and Social Brain Lab BCN NIC UMC Groningen and Netherlands Institute for Neurosciences (SBL).
• Even, for the embedding scenario 3 (i.e., Graph2Img), there is a site for which the accuracy of ASD classification is lower than Sherkatghanad et al. (2020). The case is worse for the Huang method, which works better than Graph2Img for the University of Pittsburgh School of Medicine (PITT), LEUVEN, NYU, UM-1, UCLA-1, and USM. However, the average accuracy of the leave-one-site-out validation of Graph2Img (80%) is more than that of Huang et al. (2020) with 78%.
• It seems scenarios 1 and 3 are consistent with each other, but scenario 2 is different. In the sites for which scenarios 1 and 3 obtain good results, scenario 2 does not succeed. Maybe, these methods represent different features of the graph. It is predicted that their combination would reach a good classification performance. Also, Graph2Img can be combined to use their seemingly complemental advantages.

4. For the ABIDE II database, scenario 1 (AWE method) reached the best mean accuracy. The best individual site result also is dedicated to the AWE method for the KKI database.

However, the most dominant advantage of the proposed algorithm is its training time. Using a system with two Intel Xeon E5-2620 processors with 24 cores running at 2 GHz and 48 GB of RAM. As well, 1 Tesla K40 GPU with 2880 CUDA cores and 12 GB of RAM was used to accelerate training. In such a way, the entire training time took about 200 min. In Table 5, the training time of Heinsfeld et al. (2018), Sherkatghanad et al. (2020), and our proposed method is compared. This achievement is due to the dimension reduction property of the graph embedding methods, decreasing the dimensionality of the CNN input.

The results show that the proposed algorithm, using embedded vectors of connectivity graph, and the CNN classifier, outperforms the previous studies in the identification of autism spectrum disorder, from both speed and accuracy points of view.

Since the functioning of the brain is accompanied by interactions and connections between different functional areas, discrimination of healthy and autism behaviors could be done by assessment of the brain network dynamics (Kim et al., 2017). Indeed, cognitive disorders emerge because of the alteration of dynamic relationships between pairs of specific brain regions. However, we claim that a powerful learning method considering the coupling, similarity, or causality and synchronizing intensity between specific brain regions could be able to detect cognitive impairment.

For a complete comparison, we considered the literature where a functional connectivity matrix is used to discriminant between healthy and autistic subjects, based on ABIDE database, either using conventional classifiers, deep networks, or even statistical tests. The result of these comparisons is shown in Figure 5, illustrating that embedded features achieved better results than other feature extraction methods, and as well, deep neural networks hold much greater promises than conventional classifiers. In other words, since AWE (scenario 1), Graph2Img (scenario 3), and multi-parameter Node2Vec (scenario 2) algorithms gain better classification results with CNN classifier (in leave-one-site-out validation), we claim that embedded features involving the structure of the functional connectivity of brain could be more convenient in ASD detection. The classification results (in k-fold cross-validation), although not high (66 and 64% for ABIDE I and II) enough to be appropriate for clinical usage, show that there are strong alterations in brain connections during autism disorder.

**FIGURE 6:**
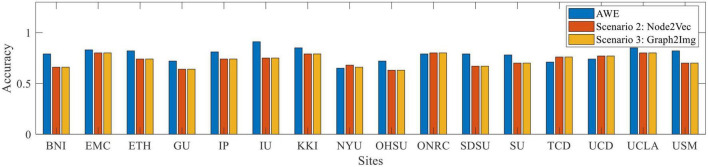
Box plot of leave-out-site accuracy to compare different embedding scenarios *vs*. sites, for ABIDE II dataset.

Our 10-fold cross-validation best average result is 66% compared to Heinsfeld et al. (2018) which is about 70%. Instead, as shown in Tables 2–4, and Figures 5, 6, for leave-one-site-out, both the mean accuracy over sites, and the most of individual accuracy of sites, our proposed method is clearly much better than Heinsfeld et al. (2018) and Sherkatghanad et al. (2020) and a little better than Huang et al. (2020). These results show that, as the sample size decreases (5-fold, 10-fold cross-validation results, and leave-one-site-out), the gap between the performance of the embedding vectors and the raw connectivity matrix increases. This implies that using embedding vectors is an effective idea, but still needs more investigation to find the more suitable graph representation method. The reason is clearly the intrinsic complexity of brain function.

## 5. Conclusion and future work

There are two messages in the obtained results: First, the intrinsic phenotypical properties of subjects within each site lead to a specific structure in their connectivity graph, in addition to the distinct indicator of ASD/healthy. Different embedding techniques acquire some of these properties. Second, a suitable combination of graph embedding techniques is the alternative approach to take all graph similarities in the ASD group regardless of the phenotypes.

The better mean accuracy of the leave-one-site-out validation technique compared to that of k-fold cross-validation again tells us about the variance of the graph structures between sites due to the within-site phenotypes. In such a way, in a random group, finding the common structures just relevant to ASD would be too difficult for an embedding technique. It is the main reason that prevents the embedding techniques capture a better result than the raw connectivity matrices.

Another interesting result is the difference in various techniques in the sites in which they can successfully detect ASD/normal situations. This point ensures us about a combinational technique, gathering all characteristics of them, to get a biomarker of ASD.

In this article, we showed that by using structural graph representation algorithms, it is possible to classify subject groups based on the connectivity fingerprints of brain regions. Therefore, our idea to use the information of node structures as a new and low-dimensional source might increase classification performance. However, such dimension reduction may lead to more ambiguity about the place of alteration in the connectivity matrix. In other words, we did not analyze the results to obtain knowledge about these alterations were of what kind, and where they occur. This is the drawback of our proposed algorithm we could not identify ROIs that alter connectivity strength values. In fact, the main point of a suitable embedding algorithm for brain network is that the representations that emerge would be neurobiologically plausible and meaningful. From this point of view, we can predict the mechanism and cause underlying an impaired brain network during mental disorders. This is our future concern.

## Data availability statement

Publicly available datasets were analyzed in this study. This data can be found here: http://fcon_1000.projects.nitrc.org/indi/abide/.

## Ethics statement

Written informed consent was obtained from the individual(s), and minor(s)’ legal guardian/next of kin, for the publication of any potentially identifiable images or data included in this article.

## Author contributions

ZM and FS conceived of the presented idea. AY performed the computations. FS verified the analytical methods and wrote the manuscript. ZM and FS encouraged AY to investigate and supervised the findings of this work. All authors discussed the results and contributed to the final manuscript.

## Footnotes

1
http://fcon_1000.projects.nitrc.org/indi/abide/
