# Supplementary material for: Detection of autism spectrum disorder using graph representation learning algorithms and deep neural network, based on fMRI signals
Source: Front Syst Neurosci. 2023 Feb 2;16:904770. doi: 10.3389/fnsys.2022.904770 (PMC9932324; doi:10.3389/fnsys.2022.904770)
Supplement: Supplementary file 1 [file Data_Sheet_1.docx]

**Appendix**

**Appendix A: Node2vec and Deep Walk**

Suppose there is a graph $G=(V,E,A)$ with $V$ as the node set, $E$ as the undirected and weighted edge set, and $A$ as the adjacency matrix. As a similarity measure, $p_{g}(v_{j}|v_{i})$ is the probability of visiting $v_{j}$ on a random walk starting from $v_{i}$ and with length $T$. For each vertex $v_{i}$, a sequence of vertices is formed, from which the probabilities can be obtained, according to the ratio of weight of edge ($v_{i}$,$v_{j}$) to the total (positive) edge weight in the $r$-truncated random walks. Here $r$ varies from 1 till the original walk length $T$. As seen in Fig. A-1, nearby nodes are mapped into vectors with small between angles.


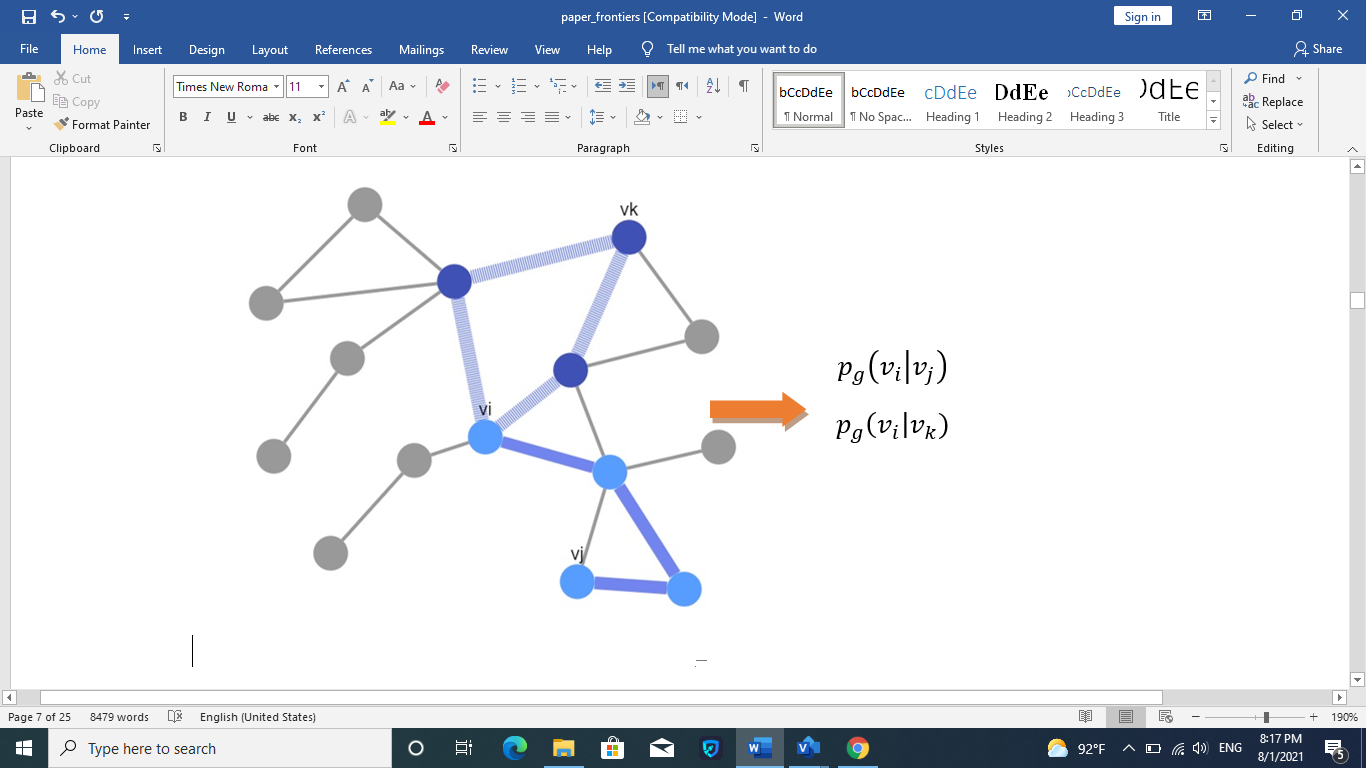

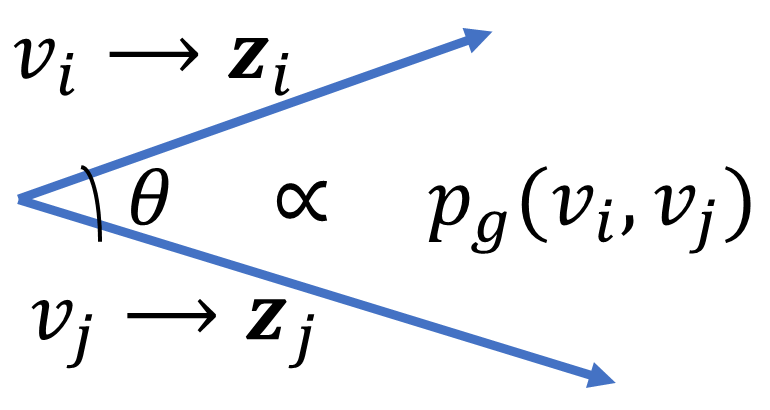


Figure A-1. Schematic of random-walk as the basis of shallow embedding techniques. Possible random walks between every two nodes lead to obtaining the conditional probability of these nodes. This probability is converted into the angle between the embedded vectors of these nodes (Hamilton William L. et al., 2017)

There are different network representation learning algorithm based on the optimization methods. But, among them Node2vec and DeepWalk are the most popular ones (Khosla et al., 2021). For DeepWalk and Node2vec algorithms, decoding is done to obtain vectors $z_{i},z_{j}$ according to an algorithm defined as below (Grover & Leskovec, 2016):

$$DEC\left( \boldsymbol{z}_{i},\boldsymbol{z}_{j} \right)\triangleq\frac{e^{\boldsymbol{z}_{i}^{T}\boldsymbol{z}_{j}}}{\sum_{v_{k}\epsilon\mathcal{V}} e^{\boldsymbol{z}_{i}^{T}\boldsymbol{z}_{k}}}\approx p_{g,T}(v_{j}|v_{i})$$

where $p_{g,T}(v_{j}|v_{i})$ is the probability of visiting $v_{j}$ on a random walk starting from $v_{i}$ and with length $T$.

Both Node2vec and DeepWalk algorithms could be easily described by the concept of context graph. This context graph is an auxiliary graph $C$ in which the weight of edge between the source node $v_{i}$, and its context $v_{j}$, *i.e.*, $c_{i,j}$ is proportional to the similarity between nodes $v_{i}$ and $v_{j}$ in graph $G$. No edge between them, is equivalent with weight −1. For a directed graph $G$, $c_{i,j}$ determines the role of a vertex as sink or source node. Based on context graph and optimization, $p_{g,T}(v_{j}|v_{i})$ is determined.

In Node2Vec and DeepWalk algorithms similarity of nodes $p_{g,T}(v_{j}|v_{i})$ is obtained via assessing the nodes sampled in random walks through graph. In other words, weights of edges of context graph $C$ is the number of walks able to reach node $v_{i}$ via $v_{j}$. Sampling the nodes on which random walk move, is different in Deep-walk and Node2vec (Khosla et al., 2021). While DeepWalk performs a uniform random walk, Node2vec follows a 2^nd^ order biased random walk. In other words, in Node2vec algorithm, negative sampling (Z. Yang et al., 2020) is used, but in DeepWalk algorithm, hierarchical softmax technique is employed. In other words, since distribution function underlying the probability of visiting a node in the random walk differ in these two techniques, the optimization cost function defined as an expectation of all combinations of elements, and subsequently the results, differs in these two algorithms.

In Fig. A-2, bread-first and depth-first search (BFS and DFS) in a graph for node $v^{*}$ (the algorithms used in random walk) are shown. In BFS the neighborhood is restricted to immediate neighbors of $v^{*}$, *i.e.*, for a neighborhood of size $k = 3$ the nodes $v_{1}, v_{2},$ and $v_{3}$. In DFS, the neighborhood consists of nodes sequentially sampled at increasing distances from $v^{*}$. In Fig. A-2, DFS samples $v_{4}, v_{5},$ and $v_{9}$.

Node2vec does an interpolation between BFS and DFS via two parameters $p \& q$, selected suitably. Hyperparameters ($p \& q$) control likelihood of walks immediately revisiting a node, and revisiting a node's one-hop neighborhood, respectively (Khosla et al., 2021). In a biased random walk, the probability of transition from a node to the next one is different according to the length of the shortest path between them (Grover & Leskovec, 2016). This can be generalized to higher order representation by considering the sequences would be appeared during random walks gathered in set $D$, including triples of nodes, with parameters $p$ & $q$ (Khosla et al., 2021). Thus, probability of traveling from node $u$ to $v$ and then $w$ indicated by $\underline{P}_{u,v,w}$ can be defined as below.

$\underline{P}_{u,v,w}=\frac{T_{u\to v\to w}}{\sum_{w} T_{u\to v\to w}}$

Where

$T_{u\to v\to w}=\left\{ \begin{matrix} \frac{1}{p} & if \left( u, v \right)\in E\left( v, w \right)\in E, u=w \\ 1 & if (u, v) \in E(v, w) \in E, u\neq w \\ \frac{1}{q} & if (u, w)\in E(v, w) \in E, u\neq w,(u, w)\notin E \\ 0 & otherwise \end{matrix} \right.$

where $E\left( v, w \right)$ is the shortest path between $v$ and $w$. This algorithm is summarized in Algorithm A-1. As well, DeepWalk algorithm is illustrated in Algorithm A-2.


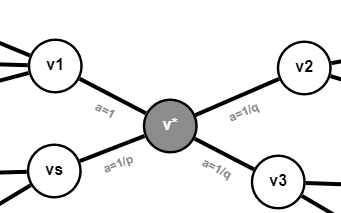

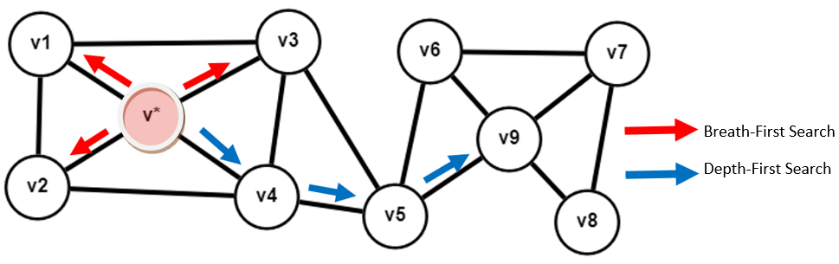


Figure A-2. A. The way Node2vec bias the random walk by p and q, B. Difference between Breath-First Search (BFS) that find one-hop neighborhood, and Depth-First Search (DFS) random walks that explore further away, to capture the structure.

Algorithm A-1. Node2Vec Algorithm (Meng & Xiang, 2018) (Grover & Leskovec, 2016)

| *Input: G(V,E)* |
| --- |
| *Walks iteration times: I* |
| *Walk length: l* |
| 1. $For iter = 1 to I do$ |
| 1. $For each node v_{i}\epsilon V$ do |
| 1. $j=0;X_{0}=v_{i}$ |
| 1. $while j<l-1 do$ |
| 1. $\{X_{j+1} is a random neigbor of X_{j} according to \underline{P}_{u,v,w}$ |
| 1. $Add node to P_{v_{i}}^{iter}=(X_{0},X_{2},\ldots,X_{j+1})$ |
| 1. $j=j+1\}$ |
| 1. $For each node we have I random paths:P_{v_{i}}^{1}to P_{v_{i}}^{I} in set D$ |
| 1. $D is complete of I paths for all nodes$ |
| 1. $Form the context graph C according to number of edges between each couple of nodes$ |
| 1. $Form p_{g,T}\left( v_{j} \vert v_{i} \right) from C$ |
| 1. $Define the cost function$ |
| 1. $Use gradient descent to solve the optimization problem ***$ |

Algorithm A-2. DeepWalk Algorithm (Perozzi et al., 2014)

| *Input: G(V,E)* |
| --- |
| *Walks iteration times: I* |
| *Walk length: l* |
| 1. $For iter = 1 to I do$ |
| 1. $For each node v_{i}\epsilon V$ do |
| 1. $j=0;X_{0}=v_{i}$ |
| 1. $while j<l-1 do$ |
| 1. $\{X_{j+1} is a random neigbor of X_{j} according to a uniform distribution$ |
| 1. $Add node to P_{v_{i}}^{iter}=(X_{0},X_{2},\ldots,X_{j+1})$ |
| 1. $j=j+1\}$ |
| 1. $For each node we have I random paths:P_{v_{i}}^{1}to P_{v_{i}}^{I} in set D$ |
| 1. $D is complete of I paths for all nodes$ |
| 1. $Form the context graph C according to number of edges between each couple of nodes$ |
| 1. $Form p_{g,T}\left( v_{j} \vert v_{i} \right) from C$ |
| 1. $Define the cost function$ |
| 1. $Use gradient descent to solve the optimization problem$ |

**Appendix B: Detail of Struc2vec**

In the struc2vec algorithm, after embedding the graph nodes into a $d$-dimensional space, representations of nodes are gathered in a matrix of dimension $N\times d$, where $N=\left| V \right|$, *i.e.*, number of nodes in the graph. Next, we can decide whether all features are important or not, and determine their priority. In fact, we can use just the most important dimension, the second important one, and so on. Here, principal component analysis (PCA) method is used to transform the $d$-dimension vector of node $v_{i}$ into dPCA-dimension vector LPCA, which is a sequential list.

$$LPCA_{i}=\left\{ f_{1i},f_{2i},\ldots,f_{dPCA,i} \right\} 1\leq i\leq N, dPCA<d$$

where $N$ represents the number of nodes in the graph. In such a way, for each component $f_{1}$ there are a $N$-dimension vector. Thus, for each pair of components, we can build a matrix of $2\times N$ dimension, and interpret it as $N$ ordered pairs, or $N$ points in a 2D plane. As an example, $M_{12}$ is built from components 1 and 2:

$$M_{12}=\left[ \begin{matrix} f_{11} & f_{12} & \ldots& f_{1,N} \\ f_{21} & f_{22} & \ldots& f_{2,N} \end{matrix} \right]$$

Digitizing values of LPCA components to $r$ equally sized bins, this two-dimensional space is partitioned into $r\times r$ bins, *i.e.*, there are $r\times r$ potential states for an ordered pair. 2D histogram of these ordered pairs (namely $H$), is a $r\times r$ matrix, in which $H_{ij}$ denotes the count of node falling into $(i,j)$ state.

Algorithm A-3. Graph2Imag Algorithm

| *Input:*  *G(V,E)* |
| --- |
| *Graph node representation, obtained via Node2Vec*  *Output:*  *Two matrices as image* |
|  |
| 1. $representations of nodes are gathered in a matrix of dimension N\times d, where N=\left\vert V \right\vert$ |
| 1. $Apply PCA method to transform the d-dimension vector of node v_{i} into dPCA-dimension vector LPCA$   $LPCA_{i}=\left\{ f_{1i},f_{2i},\ldots,f_{dPCA,i} \right\} 1\leq i\leq N, dPCA<d$ |
| 1. $Build M_{12} from components 1 and 2 of f$   $M_{12}=\left[ \begin{matrix} f_{11} & f_{12} & \ldots& f_{1,N} \\ f_{21} & f_{22} & \ldots& f_{2,N} \end{matrix} \right]$ |
| 1. $Build M_{34} from components 3 and 4 of f$   $M_{34}=\left[ \begin{matrix} f_{31} & f_{32} & \ldots& f_{3,N} \\ f_{41} & f_{42} & \ldots& f_{4,N} \end{matrix} \right]$ |
| 1. $Digitize elements of M_{12} and M_{34} to r equally sized levels, i.e., r*r bins$ |
| 1. $Obtain 2D histogram \left( H_{12} and H_{34} \right) of the ordered pairs, as a r\times r matrix$ |
| 1. $These two histograms are the Images$ |
|  |

Among all dPCA*dPCA matrices $M$, could be built by this procedure, we just consider $M_{12}$ and $M_{34}$, *i.e.*, taking into account just four first components of LPCA, which seems to be enough to analyze the brain network. As shown in Fig. [3](https://www.frontiersin.org/articles/10.3389/fncom.2018.00095/full#F3) (Meng & Xiang, 2018) these two matrices, behaving like images, can be applied as different channels of DCNN. The algorithm pseudo-code is shown in Algorithm A-3 (Meng & Xiang, 2018).

**Appendix C: AWE**

AWE is defined on weighted directed graphs. Given a weighted graph $G=(V,E,A)$, a random walk graph could be constructed $R=(V,E,P)$ in which every edge $e=(u,v)$ has a weight $p_{e}$ (Ivanov & Burnaev, 2018). A random pair of nodes $(u_{i},u_{i+1})$ can be led to a random walk with probability of $p_{e}(u_{i},u_{i+1})$. Accordingly, it is possible to attribute $p(w)$ to each random walk; as the multiplication of probability of choosing total pairs in that walk, *i.e*., $p(w) = \prod_{e\in w} p_{e}$.

Starting from each node, we can obtain marginal probability of selecting a special walk according to

$p\left( w \right)$. The probability of seeing anonymous walk $a_{i}^{u}$ of length $l$ for a node $u$ is $p\left( a_{i}^{u} \right)=\sum_{\begin{aligned} w\epsilon W^{u} \\ w\mapsto a_{i}^{l} \end{aligned}} p(w)$. where $W^{u}$ is the set of all walks starting from $u$, and $w\mapsto a_{i}^{l}$ involves the walks with length $l$. Anonymous walk embedding of this set of all possible anonymous walks with length $l$ in graph $G$ is a vector whose $i$th component is the probability of that walk. Aggregating probabilities across all vertices in a graph and normalizing them by the total number of nodes *N*, we get the probability of choosing anonymous walk $a_{i}$ in graph $G$:

$$p\left( a_{i} \right)=\frac{1}{N}\sum_{u\in G} p(a_{i}^{u})=\frac{1}{N}\sum_{u\in G} \sum_{\begin{aligned} w\in W_{l}^{u} \\ w\to a_{i} \end{aligned}} p(w)$$

AWE algorithm includes computation the probability of all random walks (Ivanov & Burnaev, 2018). Since computational cost of AWE, which requires determining all different random walks in graph $G$, grow exponentially with the number of steps $l$, the AWE algorithm is as follows (Ivanov & Burnaev, 2018):

1. Beginning from each node $u$, sample $N$ random walks starting from $u$, and map it to an anonymous random walk.
2. Gather all these sampled anonymous random walks for all nodes. These are collection of co-occurring walks.
3. Learn representation vector of these walks, and generate a $\eta*da$ dimensional matrix ($W$), where $\eta$ is the number of all possible anonymous walks of length $l$, and $da$ is the embedding size. This model is used to predict a target vector for anonymous walks in a graph.
4. For a graph vector $d$, according to sampled co-occurred anonymous walks in $W$, the model calculates a probability function to predict a target walk among all sampled anonymous walks
5. Now model updates the matrix $W$ and graph vector $d$ via gradient backpropagation, as schematically is shown in Fig. A-3.
6. After repeating steps 4 and 5, a learned graph vector d is called anonymous walk embedding.

Figure A-3. Schematic illustration of learning structure via anonymous random walks (Ivanov & Burnaev, 2018)

**Appendix D: Computing the covariance tangent space**

Nilearn Python library provides the tangent-space parametrization of covariance matrices. In this appendix it is described how this parametrization is computed, with required formulation (Dadi et al., 2019). The algorithm is made of two steps: First covariance matrix of BOLD signal of each subject is computed, and the group average matrix over all subjects are obtained. Second, this average matrix is used to transform covariance matrix to a space where ensures a well-conditioned connectivity matrix.

To estimate the average covariance matrix, the Ledoit and Wolf (Ledoit & Wolf, 2004) estimator is a good choice (Brier et al., 2015; Varoquaux & Craddock, 2013). This namely Frechet mean is calculated according to the geometry of covariance matrices (Pennec et al., 2006; Varoquaux et al., 2010), in order to minimize a cost function. This cost function is depicted in algorithm 3 of Fletcher and Joshi (Fletcher & Joshi, 2007). But, Euclidean mean with the following formula give almost the same performance.

Euclidean mean ∶ $\Sigma_{\star} =\frac{1}{n\_train}\sum_{i\in train} \Sigma_{i}$

After computing this average matrix $\Sigma_{\star}$, covariance matrix should be transformed in the tangent-space representation. This is done by first normalizing the covariance matrix of each subject, and then mapping it to a suitable space.

1. After eigenvalue decomposing of $\Sigma_{\star}=U^{T}\Delta U$, where $U$ contains eigenvectors and $\Delta$ is a diagonal matrix containing eigenvalues, we can compute the normalized matrix of each BOLD signal via

$$\tilde{\Sigma}_{i}\leftarrow U^{T}\Delta^{-1/2}U\Sigma_{i}U^{T}\Delta^{-1/2}U$$

2. After eigenvalue decomposing of $\tilde{\Sigma}_{i}=\tilde{U}^{T}\tilde{\Delta}_{i}\tilde{U}$, we can compute the matrix logarithm ${logm\tilde{\Sigma}}_{i}=\tilde{U}^{T}log(\tilde{\Delta}_{i})\tilde{U}$, where the logarithm is applied to the diagonal elements of $\tilde{\Delta}_{i}$.

Finally, the ${logm\tilde{\Sigma}}_{i}$ is the resulting matrix, and can be considered as the tangent connectivity matrix.
